# Functional Outcome of Distal Radial Fractures in Patients With a Mean Age of 75 Years at a Mean Follow-Up of 5.4 Years

**DOI:** 10.7759/cureus.11959

**Published:** 2020-12-07

**Authors:** Pranav Mishra, Mohammad Iqbal, Adnan Faraj

**Affiliations:** 1 Trauma and Orthopaedic Surgery, Scarborough Hospital, Scarborough, GBR; 2 Trauma and Orthopaedics, University Hospitals Leicester, Leicester, GBR; 3 Orthopaedics and Trauma, Scarborough Hospital, Scarborough, GBR

**Keywords:** wrist fractures, long-term outcome, plate, k-wire, frykman, prwe

## Abstract

Purpose

Distal radial fractures in the elderly are common and present in a wide spectrum of severity. Their management is varied. The aim of this retrospective case review is to evaluate the late functional outcome of surgically treated distal radial fractures in the elderly population.

Methods

Forty-two patients (36 female and six male) were surgically treated for an unstable distal radial fracture. The mean age of the patients was 75 years. Frykman classification was used to assess the severity of the injury. Surgical options used were reduction and K wires (19 patients) or open reduction and internal fixation (ORIF) using volar distal radial plate fixation (23 patients). At a mean follow-up of 5.4 years, a validated questionnaire (Patient Rated Wrist Evaluation-PRWE) of the functional ability was completed for each patient.

Results

The outcome in both groups was satisfactory (PRWE 40-50) with no significant statistical difference, however, a better functional outcome (<40 PRWE) was achieved in the K wire group compared to the ORIF group. Factors such as post injury fear from fall, weakness of grip, wrist pain, and other comorbidities altered the predicted functional outcome score.

Conclusion

In conclusion, surgically treated fractures in the elderly generally lead to good outcomes. However, confounding factors can contribute to unpredictable results despite good surgical reduction and fixation.

## Introduction

The use of internal fixation for unstable distal radial fracture is increasingly becoming popular [1,2]. Conservative treatment, however, remains a reasonable option [3]. The evidence for improved outcomes using more invasive volar locking plates in distal radial fractures in the elderly population is not robust [4-6].

Distal radial fracture characterization and treatment is mainly based on radiographic measurements. Unfortunately, evidence is lacking to correlate acceptable radiographic measurements to clinical outcomes [7]. However, there is evidence that poor radiological measurements can be associated with poor clinical results [8].

There is conflicting evidence in the current literature regarding the ‘acceptable’ radiological indices for the surgeon and correlation with functional outcomes, in patients with a displaced distal radial fracture [8,9]. This is due to the wide spectrum of injury patterns sustained, different methodologies used by the investigators, and the number of potential parameters studied. The ultimate aim of treatment remains to be a pain-free, mobile wrist joint without functional limitation [9].

The objectives of the current study were to review the long-term functional outcomes of distal radial fractures in the elderly population and correlate these results with the type of surgical intervention and early acceptable radiographic reduction according to the fracture.

## Materials and methods

This retrospective case cohort studies 42 sequential active patients with displaced distal radius fractures, managed in a district hospital between January 2011 and January 2015. ‘Active patients’ is defined as patients who are able to participate in social, economic, cultural, spiritual and civic affairs. Patients excluded from this study were: inactive, patients with dementia, those who sustained open distal radial fractures and associated carpal bone injuries, and those with ipsilateral upper limb injury. Patients with deficient medical or radiological records were also excluded. We obtained permission from the ethical department in our hospital before proceeding. The age group included in this study, were elderly patients with a mean age of 75 years (range: 60-90 years). According to the United Nations (UN), the agreed cut-off is 60+ years, to refer to the elderly.

The patients were identified using the electronic database of the hospital. There were 36 female and six male patients. Of these, 29 patients were retired and 13 patients had sedentary jobs, with occasional recreational sports activities. The left non-dominant hand was involved in nine patients, in one patient the fracture affected the dominant left wrist. The remaining patients had right-sided fractures and they were right handed. Three patients had associated lower limb injuries, two of these patients had it on the same side. The mechanism of injury was a fall in 39 patients and following road traffic injuries in the remaining three patients.

The posterior anterior (PA) radiography was taken with the shoulder abducted and the elbow flexed to 90 degrees. The lateral radiographs were taken with the shoulder adducted. The radiographs were checked by the three authors, independently.

The initial management of all these patients was an attempted closed reduction, in the emergency department. The failure of satisfactory closed reduction was considered to be an indication for surgical intervention. The operation was carried out under general anaesthesia, guided by C-arm imaging. Following closed reduction of the fracture, either Kirschner wires (K-wires) or volar plates were used for the fixation, at surgeon’s discretion. There was a tendency to use plate fixation for distal radial fractures with severe comminution and intra-articular involvement. K-wires were inserted percutaneously, using 1-2 radial styloid wires and one through dorsal ulnar distal radius following which forearm plaster immobilization for a period of 5-6 weeks was advised. The K-wires were removed in the outpatient department. Volar plate fixation was performed using acumed instruments (Smith and Nephew, London, UK). Postoperative forearm immobilization in this group was kept for a 10-day period, followed by immobilization using wrist splint for 2-4 weeks, allowing intermittent wrist exercises. Referral to physiotherapy for rehabilitation was made upon patients request (13/42 patients).

Radiological assessment - An unacceptable radiological result was considered to be

1. a gap or a step of 2 mm on PA or lateral view [8].

2. ulnar variance of more than 2 mm on the lateral view [8].

3. dorsal/palmar tilt beyond 0° (criteria used by Ng and McQueen) [8].

All these patients had intra-operative C-arm radiographs and radiographs at six weeks, following surgery.

Final telephone-based functional assessment was performed using the Patient-Rated Wrist Evaluation (PRWE) questionnaire. PRWE is a 15-item questionnaire designed to measure wrist pain and disability in activities of daily living.

In this study, the functional rating was plotted against the radiological reduction. The higher the score, the worse the function is. Scores less than 40, were considered to be good scores. Scores, between 40-50, were considered satisfactory/fair and poor scores were scores higher than 50.

## Results

A total of 42 patients were included in this study (n = 42), with completed PRWE questionnaires. The mean follow-up period for the PRWE assessment was five years and four months (range: three years and two months to seven years and six months). Statistical analysis using confidence interval (CI) -4.5 to 1.8 (p-value of 0.4), showed no significant statistical difference in the two groups, despite the slight superior result in the K-wire patient group. The period of immobilization was however longer in the K-wire cohort. Interestingly, not all the patients with good radiological results in either group, necessarily obtained a good functional outcome and vice versa.

Complications

There were four cases of loose wires in the wire group with superficial infection in two patients. There was however impaction and shortening of the radius compared to initial reduction radiographs. This did not undermine the quality of reduction and remained acceptable, according to Ng and McQueen criteria, as explained in the methods section of the paper [8]. There was no failure in reduction on radiographic examination of the plate group. None of these patients had the plates removed at the latest follow-up.

Five patients developed pain and stiffness of the wrist - three patients in the plate group and two patients in the K-wire group. Updated radiographs of the wrist at latest follow-up showed signs of secondary osteoarthritis (shortening of the radius and residual intra-articular step).

The correlation between radiological reduction and fixation, with late functional outcome, was analysed for the different genders, and types of the fracture were analysed as follows:

1. Gender:

Among the female patients (36), 17 patients underwent K-wire fixation and the remaining had volar locking plates. Out of these, 12/17 patients in the K-wire group had mean 35 (30-41) PRWE score but only 7/19 patients undergoing plate fixation had a good functional outcome, mean PRWE score of 36 (35-40).

Among the male group, the PRWE score was better in the plate group (three out of four patients) as compared to one in two patients in the K-wire group.

2. Correlation of Frykman classification and functional outcome:

The mean functional score in female patients with Frykman VIII was good, when K-wires were used for six of them and the mean functional score when plate was used for four patients, was poor. The functional score in two male patients for Frykman VIII using plate fixation, was good.

The scores for Frykman VII for patients having K-wire fixation in the female group, four scored fair and two (one patient had ipsilateral intertrochanteric fracture caused by the same fall) scored good; while the ones who had plate fixation, seven patients, also scored in the good category.

The functional score for Frykman V in a female patient using K-wire, was good.

The functional scores for Frykman IV regardless of gender (three male and three female) and method of fixation, were good (one patient treated using K-wires, and five patients using plate fixation).

The scores for Frykman III fractures, female patients, were good for those who were treated with K-wire fixation, four of them, and fair for four patients having plate fixation. There was one female patient with Frykman III fracture treated using plate fixation who scored poor (she developed a stroke on the other side and one of the screws was prominent).

One male patient had a Frykman I fracture which was treated with plate fixation and he had a good outcome. The results are depicted in the tables below (Tables 1, 2).

**Table 1:** Fracture classification and patient demographics. M: Male, F: Female

Frykman Classification	Volar-Locking Plate	K-Wire Fixation
II	1 M	0
III	5 F	4 F
IV	2 M, 3 F	1 M
V	0	1 F
VII	7 F	6 F
VIII	4 F, 2 M	6 F

**Table 2:** Results of the study. PRWE: Patient Rated Wrist Evaluation.

Score (PRWE)	Volar-Locking Plate	K-Wire Fixation
Good (<40)	10 (23.8%)	13 (31%)
Fair/Satisfactory (40-50)	4 (9.5%)	4 (9.5%)
Poor (>50)	10 (23.8%)	1 (2.4%)

## Discussion

The current paper studies the outcome of distal radial fractures treated surgically, in the elderly. The debates in this age group are influenced by what is reported in literature for younger patients with distal radial fractures [10,11]. In the UK distal radius acute fracture fixation trial (DRAFFT) study, a randomized controlled trial comparing K-wires and plate fixation, both groups of patients recovered wrist function by 12 months with no clinically significant difference [12]. In another comparative study, patients aged between 50-70 years were treated with either K-wires or volar plate fixation and the functional outcome was similar [13]. The loss of reduction when K-wires fixation is employed in the elderly aged over 75 years, is reported to be as high as 24% [14,15]. This incidence in a similar age group treated with a volar fixed-angle plate, is lower [2].

It is unclear whether maintaining good radiological reduction is associated with a good functional outcome, in the elderly. A comparative study of non-operative treatment with volar-locking plates in 70 patients aged over 70 years, showed significantly better radiological outcomes in patients treated operatively. Despite this, there was no clinically significant difference in functional outcomes [14]. In a multicentre prospective study, comparing internal plate fixation against conservative management and plaster application in displaced articular distal radius fractures, it was concluded that there is still no conclusive evidence on the optimal treatment of these fractures, in low-demand patients older than 70 years, and the outcomes were similar in both groups [2]. There is paucity in literature on the use of K-wires in the management of distal radial fracture in the elderly. Despite the absence of literature supporting the use of K-wire fixation in osteoporotic distal radial fractures, the successful use of percutaneous K-wire fixation in osteoporotic proximal humeral fractures has been published [16].

The functional outcome in the current series comparing K-wire fixation, compared to osteosynthesis of distal radial fracture in patients over 75 years of age, was similar. The maintenance of the radiological reduction is better predicted with plate fixation, however, good functional results correlated to this in only 50% of the patients. This is in contrary to the pessimism in the use of K-wire fixation. The belief has been that K-wire fixation does not provide enough purchase on osteoporotic bone. In severely comminuted or osteoporotic fractures, the trabecular bone of the metaphysis provides little inherent stability. These fractures were considered a contra-indication to percutaneous pin fixation [15]. We believe that the radiological reduction in the K-wire group was maintained by the longer period of plaster immobilization recommended.

The mean follow-up period of the functional outcome in the current paper was over five years, the functional outcome was good in 70% of the K-wire group and fair (PRWE of 50) to poor (PRWE of 65) despite good radiological outcome in 30% of the K-wire group. In more than 50% of cases in the plate fixation group, the outcome was poor (PRWE of 70) to fair (PRWE of 45). Ironically, good functional score was obtained in patients with poor radiological reduction in 15% of the K-wire group, and 13% of the plate group.

In the current study, the poor results were related to pain, weakness of the grip and fear of fall. Radiologically, ulnar variance and inflammatory arthritis (in five patients) was associated with poor functional outcome. In two patients with good radiological reduction and maintenance after plating of the radius, the functional outcome was poor because of weakness of grip. Long-term functional outcome of the management of distal radial fractures in elderly patients does not seem to correlate well with the accuracy of the radiological reduction, especially when volar plate fixation is used, as noticed in this study. Good functional outcome was better correlated to satisfactory reduction and fixation using K-wires.

The cost associated with and invasiveness of plate fixation has to be taken into consideration. The complication of volar plate fixation is reported to be high. In a study involving 114 patients, treated with volar plate fixation for distal radial fractures, the overall complication rate was 27%. The most frequent problems encountered were flexor and extensor tendon irritations (57% of the complications), including tendon ruptures [17]. Carpal tunnel syndrome (CTS) was observed in three cases, and complex regional pain syndrome (CPRS) occurred in five cases. Delayed fracture union was reported in three of the patients [17].

Moreover, K-wire fixation represented a cost-saving intervention (-£727, 95% CI -£588 to -£865), particularly when used in younger patients [17].

The limitation of this study was the inability to review the patient at the latest follow-up period, due to financial restraints. Unfortunately, the outcome measure was a telephone-based patient-rated wrist disability questionnaire. Because of the drawback of not being able to clarify the causes of the bad results, functional assessment was estimated to the best of our ability.

This study’s strength is that a validated functional outcome of the patients was obtained at mean period of over five years.

## Conclusions

At a mean follow-up of over five years of elderly patients managed for displaced distal radial fractures, long-term functional outcome was acceptable. Good reduction of the fracture was often associated with good functional outcome, but this was not always the case as described. The functional outcome of the K-wire fixation in the surgical group was superior to plate fixation.

We believe this is due to the fact that the procedure is not as invasive and manages to restore the radiological parameters following distal radial fractures well. Unpredictable confounding factors were fear of falling and weak grip. Further prospective research is required to better delineate if there are specific radiographic injuries, or patient characteristics that may benefit from volar locking plates in the short term and whether there are any differences in long-term outcomes and complications. In particular, it would be helpful to set criteria for acceptable radiological correction of wrist fractures in the elderly population.
